# YAP activation promotes the transdifferentiation of cardiac fibroblasts to myofibroblasts in matrix remodeling of dilated cardiomyopathy

**DOI:** 10.1590/1414-431X20187914

**Published:** 2018-11-23

**Authors:** Bo Jin, Jun Zhu, Hai-Ming Shi, Zhi-Chao Wen, Bang-Wei Wu

**Affiliations:** Department of Cardiology, Huashan Hospital, Fudan University, Shanghai, China

**Keywords:** YAP, Fibroblast, Cardiac remodeling, Dilated cardiomyopathy

## Abstract

Yes-associated protein (YAP) is an important regulator of cellular proliferation and transdifferentiation. However, little is known about the mechanisms underlying myofibroblast transdifferentiation in dilated cardiomyopathy (DCM). We investigated the role of YAP in the pathological process of cardiac matrix remodeling. A classic model of DCM was established in BALB/c mice by immunization with porcine cardiac myosin. Cardiac fibroblasts were isolated from neonatal Sprague-Dawley rats by density gradient centrifugation. The expression levels of α-smooth muscle actin (α-SMA) and collagen volume fraction (CVF) were significantly increased in DCM mice. Angiotensin II (Ang II)-mediated YAP activation promoted the proliferation and transdifferentiation of neonatal rat cardiac fibroblasts, and this effect was significantly suppressed in the shRNA YAP + Ang II group compared with the shRNA Control + Ang II group *in vitro* (2.98±0.34 ×10^5^ *vs* 5.52±0.82 ×10^5^, P<0.01). Inhibition of endogenous Ang II-stimulated YAP improved the cardiac function by targeting myofibroblast transdifferentiation to attenuate matrix remodeling *in vivo*. In the valsartan group, left ventricular ejection fraction and fractional shortening were significantly increased compared with the DCM group (52.72±5.51% *vs* 44.46±3.01%, P<0.05; 34.84±3.85% *vs* 26.65±3.12%, P<0.01). Our study demonstrated that YAP was a regulator of cardiac myofibroblast differentiation, and regulation of YAP signaling pathway contributed to improve cardiac function of DCM mice, possibly in part by decreasing myofibroblast transdifferentiation to inhibit matrix remodeling.

## Introduction

Dilated cardiomyopathy (DCM), characterized by a loss of cardiomyocytes and accumulation of extracellular matrix, is an important cause of congestive heart failure (1–3). Most investigations of mechanisms underlying cardiac function focus on structural changes in cardiomyocytes to explain the deleterious contractile function (4,5). However, alterations in the extracellular matrix of myocardium are also recognized to play important roles (6,7). The exact mechanism of fibroblast transdifferentiation to myofibroblasts is still unclear. Here, we explored the distinct role of Yes-associated protein (YAP) in the transdifferentiation of myofibroblasts during the pathological process of cardiac matrix remodeling.

The activity of the sympathetic nervous system has been reported to be increased in both humans and animals with chronic heart failure. One of the mechanisms believed to be responsible for this phenomenon is an increased systemic angiotensin II (Ang II) signaling (8,9). Moreover, in a previous study, conversion of resting fibroblasts to pro-fibrogenic myofibroblasts in response to Ang II and endothelin resulted in cardiac fibrosis (10). Hippo signaling pathway plays important roles in the control of organ size, tissue homeostasis, and regeneration, and dysregulation of the Hippo/YAP pathway can lead to uncontrolled cell growth and malignant transformation. The Hippo/YAP pathway can be both tumor suppressive and oncogenic. This will be crucial before anti-cancer therapies targeting this pathway can be implemented (11). As part of the Hippo signaling cascade, YAP was shown to govern organ size by influencing cell proliferation and cell death in both Drosophila and mammals (12). We hypothesized that YAP signaling pathway is the barrier preventing the proliferation and transdifferentiation of cardiac fibroblasts. Regulation of YAP signaling pathway by targeting transdifferentiation to inhibit matrix remodeling can improve cardiac function in DCM.

The aim of this study was to elucidate whether and how YAP plays a role in myofibroblast differentiation in the context of Ang II and the pathophysiology of DCM. The study will not only contribute to clarify the pathophysiological mechanism, but also may provide novel strategies for clinical treatment of DCM.

## Material and Methods

### Experimental model of DCM

The animal studies were approved by the Animal Care and Utilization Committee of Fudan University. The experimental model was established in BALB/c mice by immunization with porcine cardiac myosin (Sigma, USA) to induce DCM (13). Cardiac myosin was emulsified with an equal volume of complete Freund's adjuvant (Sigma) to a final concentration of 5 mg/mL. The solution was then subcutaneously injected into the groin of BALB/c mice at days 0 and 7, as previously described (14). The control group was treated with Freund's adjuvant alone. The study included the following three experimental groups: Control group, DCM group, and valsartan-treated group. The DCM mice of the valsartan-treated group were treated with 8 mg·kg^-1^·day^-1^ valsartan (Novartis, Switzerland) for 4 weeks by oral gavage.

### Echocardiography

M-mode echocardiography is considered the most effective and safe method to measure cardiac chamber size and cardiac function. Thus, 8 weeks after immunization, transthoracic echocardiography was performed using a 7.5-MHz imaging transducer (Philips Medical System, Netherlands). The mice were anesthetized and their chests epilated. M-mode images were obtained at the level of papillary muscles in the long-axis view. Left ventricular end-diastolic dimension (LVEDD), left ventricular end-diastolic volume (LVEDV), fractional shortening (FS), and left ventricular ejection fraction (LVEF) were measured.

### Neonatal rat cardiac fibroblast isolation

Neonatal rat cardiac fibroblasts were isolated from 1-3 day old Sprague-Dawley rats as previously reported (15). Briefly, ventricles were minced and digested 3 times in 0.3 mg/mL collagenase II. The hearts and fluid were incubated in a 37°C shaker oven for 10 min. Cell suspensions were collected and combined for Percoll density gradient centrifugation to separate cardiac fibroblasts. Three milliliters of the 1.082 g/mL Percoll (Sigma) was pipetted into separate sterile 15-mL conical tubes and 3 mL of the 1.062 g/mL Percoll was layered on top of the bottom layer. Then, 3 mL of the 1.050 g/mL Percoll was layered on top. The tubes were spun at 1500 *g* in a tabletop centrifuge (Thermo Fisher, USA) for 30 min at room temperature starting slowly using no brake. The fibroblasts were pipetted from the top of the 1.050 layer into a sterile tube. Purified fibroblasts were seeded in DMEM containing 15% fetal bovine serum (FBS). After overnight attachment, the medium was replaced with a solution containing 100 U/mL penicillin, 100 µg/mL streptomycin, and 250 ng/mL amphotericin B and maintained throughout cultures.

### shRNA-mediated YAP knockdown in cardiac fibroblasts

Stable gene knockdown was performed using lentiviral shRNA targeting YAP. Target sequence of YAP was obtained from the MISSION®shRNA library (Sigma-Aldrich, USA) and packaged into lentiviral particles (16). Used sequence was as follows: 5-CCGGTGAGAACAATGACAACCAATACTCGAGTATTGGTTGTCATTGTTCTCATTTTTG-3. Scrambled sequence was as follows: 5-CCGGGTACTGATGTCGAAAGTAGACCTCGAGGTCTACTTTCGACATCAGTACTTTTTC-3. YAP shRNA-transfected and control shRNA-transfected cardiac fibroblasts were cultured in 6-well plates. After transfection for 48 h, cardiac fibroblasts were harvested for viability and cell cycle analysis. The efficacy of transfection was confirmed by western blotting (Supplementary Figure S1), as previously described (17).

### Histopathology

After sacrifice, the mouse hearts were fixed in 4% formaldehyde, embedded in paraffin, and cut into 5-μm thick sections. Specimens were stained with sirius red, and microscopic images were evaluated. Collagen volume fraction (CVF) was determined by quantitative morphometry of specimens with IMS Cell Image Analysis System (Shanghai, China). Five random fields of view were examined for CVF analysis across the left ventricular section.

### MUSE cell analyzer to assess cell cycle

Neonatal rat cardiac fibroblasts were resuspended in PBS and added in drops into a tube containing 1 mL of ice-cold 70% ethanol. The samples were stored at −20°C for at least 3 h. Subsequently, the fixed cells were resuspended in 200 μL of Muse™ cell cycle reagent and incubated for 30 minutes. After incubation, cardiac fibroblasts were analyzed by Muse™ cell analyzer (Merck-Millipore, USA) according to the manufacturer's instructions. The kit allows for easy and rapid quantitative measurements of the percentage of fibroblasts in the G0/G1, S, and G2/M phases of the cell cycle (18).

### Immunofluorescence microscopy

Neonatal rat cardiac fibroblasts were washed twice with phosphate-buffered saline (PBS) and fixed in 2% paraformaldehyde for 10 min. Cardiac ventricles were harvested, frozen, mounted on a cryostat, and cut into 10-μm sections. Fibroblasts and tissue sections were fixed in cold acetone, blocked with 4% BSA in 0.1% Tween and PBS, and incubated with primary antibodies. After incubation with Alexa-conjugated secondary antibodies and staining of nuclei with DAPI, samples were mounted in gelvatol (Beyotime, China). Primary mouse anti-human α-SMA (dilution 1:100; ab5694; Abcam, UK), mouse anti-human vimentin (dilution 1:1000; V6384; Sigma), and rabbit anti-human YAP (dilution 1:100; 4912; Cell Signaling Technology, USA) were diluted in PBS that contained 2.2% bovine serum albumin. Nuclei were visualized with DAPI (dilution 1:5000; C1002; Beyotime), mouse anti-human vimentin (dilution 1:1000; V6384; Sigma), and rabbit anti-human YAP (dilution 1:100; 4912; Cell Signaling Technology) were diluted in PBS that contained 2.2% bovine serum albumin. Images were obtained using a laser scanning confocal microscope and Zeiss Image Examiner software (Olympus, Japan).

### Western blotting and dot blotting

Proteins were extracted from the cardiac fibroblasts and myocardial tissues homogenized in RIPA Lysis (P0013B; Beyotime) and Extraction Buffer with a protease inhibitor cocktail, and proteins were quantified using the bicinchoninic acid method. Samples of 25-μg protein were loaded into 8% SDS-PAGE gels for electrophoresis then transferred to PVDF membranes overnight at 30V. Antibodies specific for α-SMA (dilution 1:500; ab5694; Abcam), YAP (dilution 1:100; 4912; Cell Signaling), p-YAP (dilution 1:100; 4911; Cell Signaling), and collagen I (dilution 1:1000; ab93095; Abcam) were incubated at 4°C overnight, and GAPDH (dilution 1:5000; sc66163; Santa Cruz, USA) was used as a loading control to normalize gel loading and protein expression. HRP-conjugated secondary antibodies (dilution 1:300; AS10 653; Agrisera, Sweden) plus ECL (AS16; Agrisera) were incubated at 37°C for 1 h for protein visualization. The densitometric values of bands were measured using densitometry analysis software (Multi Gauge Ver 3.0, Japan).

### Statistical analysis

Data are reported as means±SD. P<0.05 was considered statistically significant. Normal distribution was confirmed in all groups, and differences in means between two groups were analyzed by unpaired Student's *t*-test. Multiple group comparison was performed by one-way ANOVA followed by Newman-Keuls multiple comparison test.

## Results

### YAP was activated in the matrix remodeling of DCM

**Figure 1.**
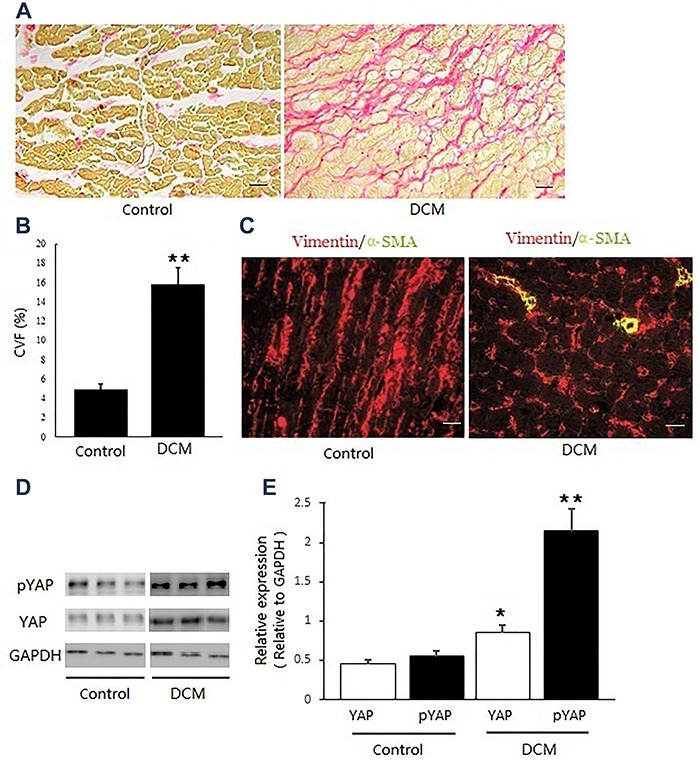
Yes-associated protein (YAP) was activated in the matrix remodeling of dilated cardiomyopathy (DCM). *A*, Picrosirius red staining indicated an increased collagen distribution in the DCM group. *B*, Quantitative assessment demonstrated the collagen volume fraction (CVF) significantly increased in the DCM group. *C*, Immunofluorescence staining of α-smooth muscle actin (α-SMA) confirmed the transdifferentiation of cardiac myofibroblasts in the pathogenesis of DCM. *D* and *E*, The protein levels of YAP and pYAP were significantly increased in the matrix remodeling of DCM. Data are reported as means±SD (n=8). *P<0.05, **P<0.01 *vs* control (*t*-test). In *D*, each lane shows protein from a different mouse. Scale bar: 100 μm.

The experimental model of DCM was established in BALB/c mice by immunization with porcine cardiac myosin. Histochemical analysis with picrosirius red staining indicated that there was a significant increase of collagen distribution (stained red) in the DCM group compared with the control group (Figure 1A). For the DCM group, CVF was significantly increased to 15.77±1.62% compared with the control group, revealing myocardial fibrosis (Figure 1B). The cardiac fibroblasts and myofibroblasts were both vimentin-positive, whereas only myofibroblasts were α-SMA-positive (19). Therefore, the status of vimentin staining in the control seemed to be similar to that in DCM. Immunofluorescence staining of α-SMA positive confirmed the transdifferentiation of cardiac myofibroblasts in the pathogenesis of DCM (Figure 1C). Furthermore, the protein levels of YAP and pYAP were significantly increased in the matrix remodeling of DCM (Figure 1D and E).

### Ang II activated YAP in cardiac myofibroblast transdifferentiation *in vitro*

**Figure 2.**
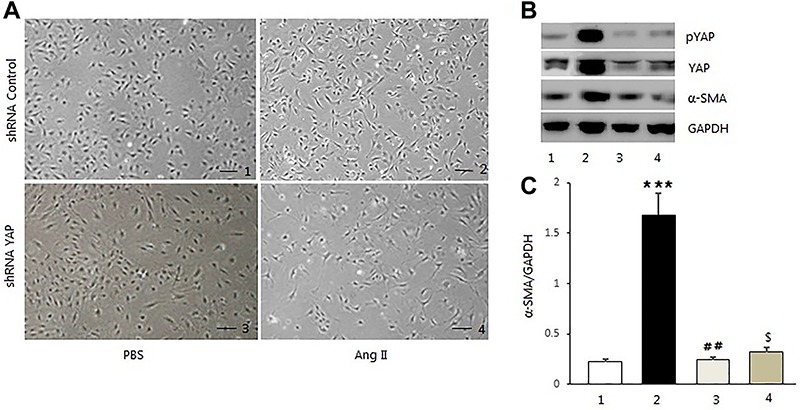
Angiotensin II (Ang II) activated Yes-associated protein (YAP) in cardiac myofibroblast transdifferentiation. *A*-*C*, The expression of YAP was significantly decreased by infecting lentiviral shRNA targeting YAP. Scale bar: 50 μm. Data are reported as means±SD. ***P<0.001 *vs* shRNA control + PBS (1); ^##^P<0.01 and ^$^P<0.05 *vs* shRNA control + Ang II (2) (ANOVA). 3: shRNA YAP + PBS; 4: shRNA YAP + Ang II. Each experiment was conducted 3 times in triplicate.

To verify whether Ang II was involved in the proliferation and transdifferentiation of myofibroblasts, neonatal rat cardiac fibroblasts were incubated with 100 nM Ang II for 24 h after serum starvation. This experiment included four groups: shRNA Control + PBS (normal group), shRNA Control + Ang II (YAP activation group), shRNA YAP + PBS (YAP knockdown + PBS group), shRNA YAP + Ang II (YAP knockdown + Ang II group). Figure 2A shows that cardiac fibroblasts were significantly increased in the YAP activation group compared to the control group (5.52±0.82 ×10^5^ *vs* 3.64±0.38 ×10^5^, P<0.01). However, cardiac fibroblast proliferation was inhibited after YAP knockdown, and Ang II activation was suppressed in shRNA YAP + Ang II group compared to the YAP activation group (2.98±0.34 ×10^5^ *vs* 5.52±0.82 ×10^5^, P<0.01). We found that YAP and pYAP protein levels significantly increased following stimulation with Ang II compared to the control group. The increased expression of α-SMA indicated transdifferentiation of cardiac myofibroblasts after Ang II treatment. To determine whether Ang II could activate YAP in cardiac fibroblasts, we decreased the expression of YAP using lentiviral shRNA targeting YAP. After infection for 48 h, cardiac fibroblasts were harvested and YAP expression was determined by immunoblot analysis. In unstimulated fibroblasts, YAP knockdown had no effect on the expression of α-SMA. Furthermore, following stimulation with Ang II, YAP-deficient fibroblasts with low YAP activity did not show an increase of α-SMA, whereas control fibroblasts showed significantly increased α-SMA expression (Figure 2B,C).

### YAP was required for cardiac myofibroblast transdifferentiation *in vitro*

**Figure 3.**
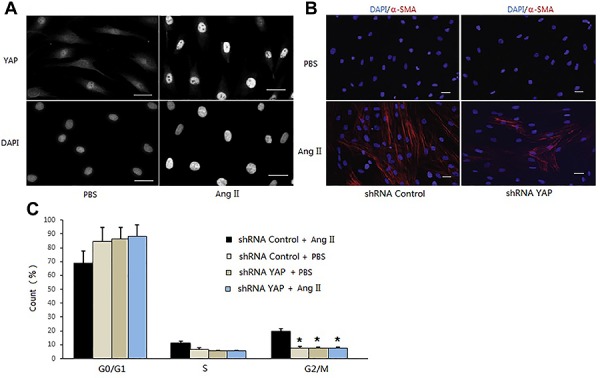
Yes-associated protein (YAP) was required for cardiac myofibroblast transdifferentiation. *A*, Immunofluorescence staining of YAP and DAPI indicated that YAP was activated as seen by its nuclear localization in angiotensin II (Ang II-stimulated fibroblasts. *B*, α-smooth muscle actin (α-SMA) expression at 24 h of Ang II stimulation was significantly lower after YAP knockdown, as shown by immunofluorescence of α-SMA (red) and DAPI (blue) staining. *C*, Cardiac fibroblast activation was inhibited by quantitative measurements of the percentage of cells in the G2/M phases of cell cycle using the Muse™ cell analyzer. Data are reported as means±SD. *P<0.05 *vs* shRNA control + Ang II (ANOVA). Scale bar: 50 μm. Each experiment was conducted 3 times in triplicate.

YAP was activated by Ang II in the transdifferentiation of neonatal rat cardiac fibroblasts. Immunofluorescence staining of YAP and DAPI indicated that YAP was weak in both the cytoplasm and nucleus of the BPS group, but on Ang II stimulation, YAP translocated to the nucleus. Our results indicated that in Ang II-stimulated fibroblasts, YAP was already activated as demonstrated by its nuclear localization in the qualitative analyses (Figure 3A). Knockdown of YAP by shRNA inhibited fibroblast proliferation, and reduced the expression of α-SMA significantly. Not having data for collagen I expression is a limitation of the present study. We performed the shRNA-mediated knockdown of YAP during Ang II-induced myofibroblast differentiation and verified that YAP was required for the phenotype transition by immunofluorescence. Interestingly, we found that α-SMA expression following 24 h of Ang II stimulation was significantly lower after YAP knockdown, as shown by immunofluorescence (Figure 3B). Cardiac fibroblast proliferation was confirmed by quantitative measurements of the cell percentage in the G0/G1, S, and G2/M phases of cell cycle. The percentage of cardiac fibroblasts in the G2/M phases was increased after Ang II stimulation, while it was significantly decreased after YAP knockdown. Furthermore, there was no change in G2/M in the shRNA YAP control group compared to the shRNA YAP + Ang II group (Figure 3C).

### Inhibition of Ang II-stimulated YAP can improve cardiac function of DCM mice

**Figure 4.**
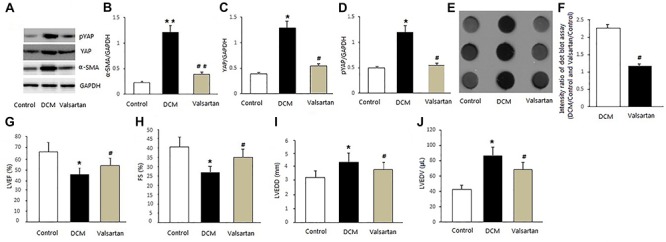
Inhibition of angiotensin II (Ang II)-stimulated Yes-associated protein (YAP) can improve cardiac function. *A*-*D*, Inhibition of inner Ang II-stimulated YAP resulted in a decrease in the protein levels of YAP, pYAP, and α-smooth muscle actin (α-SMA) in the valsartan-treated group. *E* and *F*, The expression of collagen I was significantly decreased to attenuate matrix remodeling in the valsartan-treated group compared to the dilated cardiomyopathy (DCM) group in dot blotting analyses. *G* and *H*, Cardiac function differed significantly between the groups, and the left ventricular ejection fraction (LVEF) and fractional shortening (FS) significantly improved in the valsartan group. *I* and *J*, Left ventricular end-diastolic dimension (LVEDD) and left ventricular end-diastolic volume (LVEDV) were significantly decreased in the valsartan group. Data are reported as means±SD (n=8). *P<0.05, **P<0.05 *vs* Control; ^#^P<0.01, ^##^P<0.01 *vs* DCM (ANOVA).

We next examined whether we could translate the above findings to a more clinically relevant setting in the context of DCM. Previous studies have demonstrated that activation of the inner Ang II is a key mediator of heart failure progression (20). Our experiments suggested that YAP remained active in the setting of chronic heart failure *in vivo*. Inhibition of inner Ang II-stimulated YAP resulted in a decrease in the protein levels of YAP, pYAP, and α-SMA in the valsartan-treated group (Figure 4A-D). The absence of change over time for the YAP, pYAP, and α-SMA levels is a limitation of our study. Furthermore, the expression of collagen I decreased enough to attenuate matrix remodeling in the valsartan-treated group compared with the DCM group in dot blotting analyses (Figure 4E and F). As summarized in Figure 4G and H, cardiac function differed significantly among the groups. In the DCM group, LVEF and FS significantly deteriorated compared to the control group. In the valsartan group, LVEF and FS were significantly increased compared to the DCM group, although cardiac function was still lower compared to the control group. Furthermore, LVEDD and LVEDV significantly decreased following down-regulation of YAP to inhibit cardiac matrix remodeling (Figure 4I and J). The Ang II type 1 receptor blocker valsartan, which inhibited the fibroblast-to-myofibroblast transformation, may provide a therapeutic means for preventing maladaptive remodeling, in part by down-regulation of YAP in chronic heart failure (21).

## Discussion

Transdifferentiation of cardiac fibroblasts to myofibroblasts, characterized by expression of α-SMA and production of extracellular matrix components, is a key event in cardiac matrix remodeling (22–24). To the best of our knowledge, this is the first time that YAP signaling pathway has been reported as the barrier to prevent the transdifferentiation of myofibroblasts in matrix remodeling of DCM. The effects were verified *in vitro* and *in vivo*. Our data provided evidence that YAP played a distinct role in the regulation of myofibroblast differentiation. The mechanisms underlying the transdifferentiation of cardiac myofibroblasts remain not fully understood. Ang II-stimulated YAP might play an important role in the pathology of chronic heart failure. This study suggested that YAP was a promising therapeutic target in the treatment of DCM.

The Hippo pathway has been shown to promote cell death and differentiation and inhibit cell proliferation. Several studies have demonstrated that YAP/TAZ is a candidate oncogene and that other members of the Hippo pathway are tumor-suppressive genes. The dysregulation of the Hippo/YAP pathway has been observed in cardiovascular diseases. Subsequent research identified that YAP was negatively regulated by the Hippo pathway (25). YAP is a transcriptional co-activator, known for its role in mechanobiology in several cell types (26–28). In cancer-associated fibroblasts, YAP activity is necessary for the increase of cytoskeletal components. In these cells, YAP deficiency resulted in decreased matrix remodeling and contraction (29). Previous studies suggested that YAP-deficient cells have a decreased capacity to deposit collagen, which is consistent with the results found in the present study (30–32). Consistently, we found that α-SMA protein level was decreased after YAP knockdown *in vitro*. We decided to exclusively focus on Ang II-stimulated YAP to demonstrate its role in cardiac matrix remodeling of DCM, which to the best of our knowledge, has not been previously reported.

Ang II-stimulated fibroblasts are well known for their excessive production of extracellular matrix components and the ability to remodel and contract the surrounding tissue (33,34). Stimulation with Ang II results in an increase of collagen I and maturation of the cytoskeleton. Furthermore, it remains unclear how Ang II promotes the induction of a synthetic and contractile myofibroblast phenotype (35). In the present study, YAP deficiency resulted in protective effects by decreasing the expression of α-SMA *in vitro*. YAP localized in both the cytoplasm and nucleus in cardiac fibroblasts, through regulating both YAP subcellular localization and protein stability, phosphorylation ensures a spatial and temporal control of YAP activity. Previous studies show that YAP plays an important role in the Hippo signaling cascades (36,37). Our data suggested that Ang II stimulated YAP translocation to the nucleus and induced myofibroblast transdifferentiation. The results agree with the previous study that identified YAP as a key molecule to inhibit cardiomyocytes proliferation (38). Echocardiography cannot be used to evaluate cardiac matrix remodeling. In our study, we evaluated matrix remodeling by collagen I and CVF *in vivo*. Our study indicated that inhibition of Ang II-stimulated YAP decreased the protein levels of YAP, pYAP, α-SMA, and collagen I *in vivo*. In the valsartan group, cardiac function significantly improved compared to the DCM group, although LVEF was still lower compared to the control group. Furthermore, LVEDD and LVEDV reduced by decreasing the expression of YAP to inhibit the matrix remodeling.

The phenomenon of myofibroblast formation from fibroblasts and its pro-fibrogenic role in the production of connective tissue is well conserved regardless of the tissue of residence (39). In the heart, one confronts this problem in the setting of cell injury and associated cardiac fibrosis, especially in congestive heart failure (40). It is necessary to develop a strategy to limit the continued production of extracellular matrix that can eventually lead to diminished contractile function. The Ang II type 1 receptor blocker valsartan, which inhibited the fibroblast-to-myofibroblast transformation, may provide a therapeutic means to prevent maladaptive remodeling by decreasing the expression of YAP in chronic heart failure. This study will contribute to the development of novel strategies to attenuate and prevent cardiac fibrosis of DCM.

In summary, our study demonstrated that YAP is a regulator of cardiac myofibroblast differentiation, and regulation of YAP signaling pathway contributes to improve cardiac function of DCM mice, possibly in part by decreasing myofibroblast transdifferentiation to inhibit matrix remodeling. The present study clarified the pathophysiological mechanism of DCM and provided a basis for novel strategies for clinical treatment.

## Supplementary Material

Click here to view [pdf]
